# A case of caudal regression syndrome: walking or sitting?

**DOI:** 10.11604/pamj.2014.18.92.3683

**Published:** 2014-05-26

**Authors:** Irem Bicakci, Selin Turan Turgut, Bekir Turgut, Afitap Icagasioglu, Zeliha Egilmez, Yasemin Yumusakhuylu

**Affiliations:** 1 Istanbul Medeniyet University Goztepe Training and Research Hospital, Physical Medicine and Rehabilitation Department, Istanbul, Turkey; 2 Karaman State Hospital, Physical Medicine and Rehabilitation Clinic, Karaman, Turkey; 3 Karaman State Hospital, Radiology Clinic, Karaman, Turkey

**Keywords:** caudal regression syndrome, sacral agenesis, congenital malformation, maternal diabetes, knee flexion contracture

## Abstract

Caudal regression syndrome (CRS) is a congenital disorder which is seen vertebral anomalies in varying degrees from lower thoracic spineto the level of the coccyx. We present a case of CRS which is not intended operation for orthopedic deformities considering functionality. 2, 5 year-old girl referred to our clinic with complaints about walking disability, knee and foot deformities. Patient's mother with unregulated diabetes did not have a history of drug use, radiation exposure and serious illness during pregnancy. Diagnosis had been put during antenatal follow-ups. On physical examination, her lower extremities were hypoplastic and had no muscle activity. Her hips were flexed and abducted, but did not have contractures. Her knees had 75 degrees of flexion contractures with popliteal webs and feet had equinovarus deformity. Frog belly was present due to abdominal muscles weakness. Also hypoplasic labia majora has been identified. In lumbar MRI, spinal cord was terminated at 6th thoracic (T6) vertebrae and the last solid vertebrae level was at T10. Patient who has been following by urology with clean intermittent catheterization had also severe urological problems including horseshoe kidney, neurologic bladder, vesico-ureteral reflux and grade 2 hydronephrosis. Orthopedic consultation was made for her deformities. They decided that ambulation unexpected patient's knee flexion contractures were helping sitting balance. Because of this operation was not considered. Prognosis, treatment options, strength exercises for upper extremities, skin care were told to parents and patient was taken to follow. CRS is a rare congenital abnormality which is associated with orthopedic deformities, as well as urological, anorectal and cardiac malformations. Treatment requires a multidisciplinary approach. It should not be forgotten that purpose of rehabilitation is not to correct all deformities but increase the functionality of everyday life.

## Introduction

Caudal regression syndrome is a rare congenital disorder accompanied by varying degrees of vertebrae abnormalities with an incidence of 1:7500 to 1:60.000 and may extend from the lower thoracic vertebrae to coccyx level [1, 2]. Possible risk factors include uncontrolled maternal diabetes, genetic susceptibility and vascular hypoperfusion, however the exact pathogenesis is unclear [3]. Many additional abnormalities may be observed including urologic abnormalities such as renal ectopia and agenesis, gastrointestinal system abnormalities such as imperforate anus and anorectal atresia, neural tube abnormalities such as tethered-cord, diastematomyelia, lipomyelomeningosel and congenital narrow spinal tract or orthopaedic deformities such as dysplastic vertebrae, scoliosis, hip dislocation and contracture, knee flexion contracture accompanied by popliteal webbing, narrow pelvis, syringomyelia, club foot and frog leg [1, 2, 4]. Knee flexion contractures accompanied by popliteal webbing are the most difficult orthopaedic deformities to treat. Orthopaedic interventions may vary between the minimal orthopaedic intervention and aggressive surgical intervention procedures based on the severity of deformity and targeted functional level [5]. In this study, we presented a case with caudal regression syndrome with bilateral knee flexion contractures accompanied by popliteal webbings that have not been considered for surgical intervention as they helped to sitting stability.

## Patient and observation

**Figure 1:**
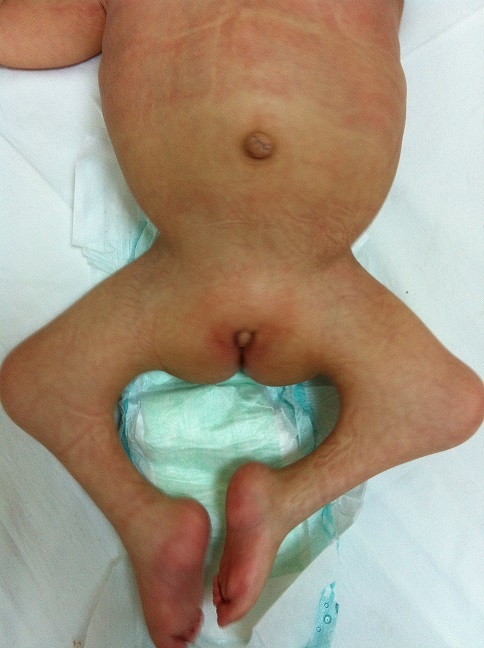
Frog abdomen, hypoplasia of labia majora, knee flexion contractures, equinovarus deformity

**Figure 2:**
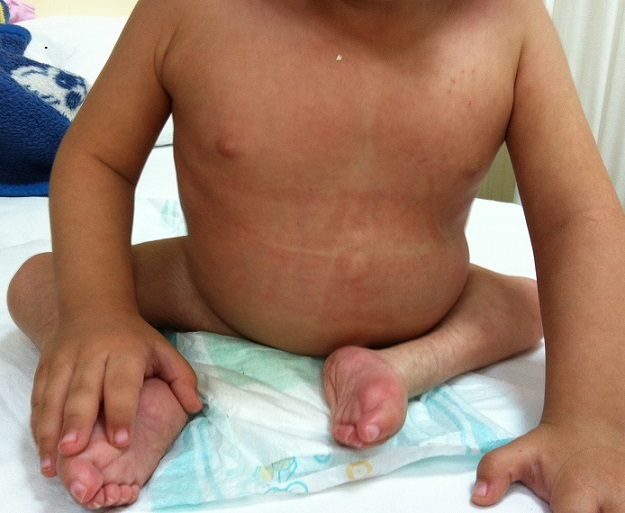
Patient sitting on a functional position

**Figure 3:**
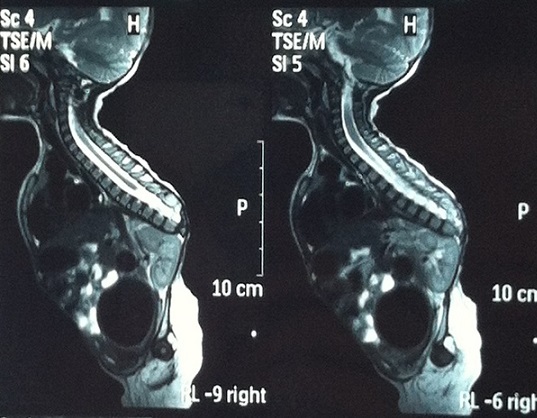
Spinal cord was observed to be terminated at T6 vertebral corpus level,lower than T10 vertebra level, thoracic and lumbar vertebrae and sacrum couldn't be observed according to lumbar MRI

**Figure 4:**
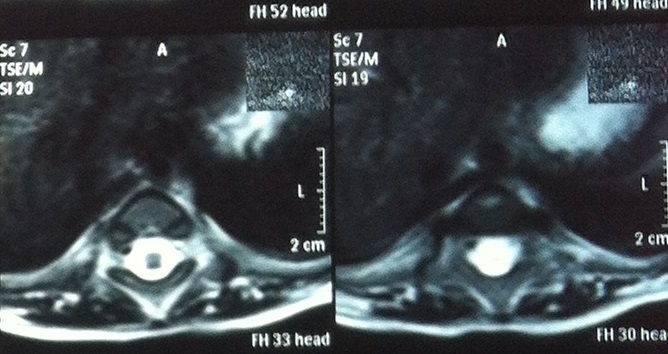
Spinal cord was observed to be terminated at T6 vertebral corpus level, lower than T10 vertebra level, thoracic and lumbar vertebrae and sacrum couldn't be observed according to lumbar MRI

**Figure 5:**
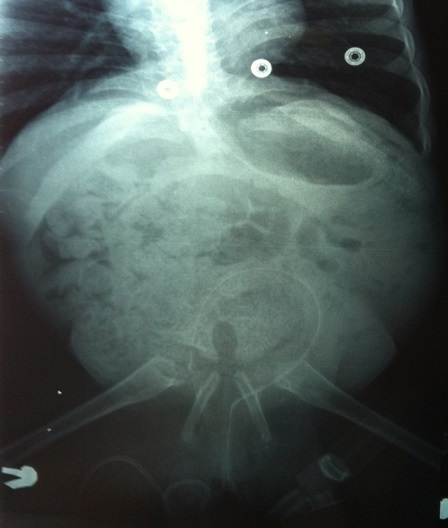
In voiding cystourethrography, total sacral agenesis and partial lumbar vertebrae agenesis were observed

A 2.5 year-old girl applied to our clinic with the complaints of abasia and deformity in knees and feet. She was the third child of a diabetic mother according to her medical history and consanguineous marriage was not the case for her parents. It was understood during the antenatal follow-up that the diagnosis has been made during 28th-gestational week and caesarean delivery has been performed at 34th-gestational week. There were no history of drug use, radiation exposure and serious disease for the mother, for whom the blood glucose has not been regulated during pregnancy. It was ascertained that there were no similar congenital deformities in other family members including the healthy siblings of patient. In locomotor system examination, lower extremities of patient were hypoplasic and there were no activity in all key muscles of lower extremities including quadriceps. Her hips were in flexion and abduction position when patient was in supine position but there were no contracture. Flexion contracture of 75 degree accompanied by popliteal web was observed in the knees of patient. Equinovarus deformity has been observed in both feet. Hypoplasia of the labia majora has been determined in genital area. No deformities were detected in upper extremities, but she had frog abdomen due to weakness of the abdominal muscles. Other system examinations were normal (Figure 1, Figure 2). Total sacral agenesis and partial lumbar vertebrae agenesis were observed in voiding cystourethrography performed due to repeated urinary tract infections and spinal cord was observed to be terminated at T6 vertebral corpus level according to lumbar MRI (Figure 3, Figure 4, Figure 5). Vertebrae were observed to the T10 vertebra level and lower thoracic and lumbar vertebrae and sacrum couldn't be observed. Tethered cord and other anomalies of vertebrae such as hemivertebrae were not detected in patient. In the urinary ultrasonography, horseshoe kidney with the fusion abnormality of both kidneys at lower pole and bilateral grade 2 hydronephrosis were observed. In the urodynamic evaluation, detrusor overactivity and grade 2 vesicoureteral reflux (VUR) were determined consistent with the neurogenic bladder. It was understood that the patient has been administered with clean intermittent catheterization with the urologic follow-up. Cervical MRI and transfontanelle USG results of the patient were normal and result of cytogenetic analysis was 46,XX normal female karyotype. Deformities of lower extremities were considered for surgery by consulting to orthopaedics department. However, the surgery was not found to be appropriate as the flexion contractures in the knees considered to help the sitting stability of the patient having no ambulation potential due to spinal-pelvic instability. Family has been informed on prognosis and treatment options, upper extremity strengthening exercises, good skin care and positioning, and follow-up procedures have been initiated for patient.

## Discussion

Caudal regression syndrome is a rare congenital disorder and its pathogenesis is not exactly known. Of the 100.000 pregnancy occurred, only 1 to 2.5 is resulted in diagnosis of caudal regression syndrome [6]. This abnormality has been reported to frequently result from neurulation defects during the first 28 days of foetal development or malformations in differentiation phase [1]. Most of the cases are sporadic. Partial genetic contribution has been reported, however, there is no proven Mendelian inheritance pattern. Recent studies showed the involvement of HLXB9 gene in occurrence of some types of sacral agenesis[6]. Although the pathogenesis is unclear, the maternal pathology having most known association is diabetes mellitus [3]. Despite the low incidence in general population, it is observed at rate of 1/350 in infants of diabetic mothers, which is a 200-fold higher incidence compared to general population. Also in this case, mother had the insulin-dependent diabetes mellitus. Anorectal and urogenital system malformations such as imperforate anus, anorectal atresia, renal and ureter agenesis, duplication and fusions may develop following the inhibition of cloaca with caudal agenesis [7]. Anorectal, genital and cardiac malformations may also accompany to sacral agenesis. Cases of delayed diagnosis with clinical evaluation and occurrence of vesicoureteral reflux, hydronephrosis and irreversible serious problems secondary to chronic urinary tract infection have been reported. While there were no anorectal and cardiac malformation in this case, horseshoe kidney formation and external genitalia hypoplasia were observed and grade 2 hydronephrosis and VUR due to neurogenic bladder were detected. These patients may also have multiple congenital neurologic and orthopaedic abnormalities [4]. Also in this case, there were orthopaedic deformities such as popliteal web and flexion contracture in both knees and equinovarus deformity in both feet. A careful evaluation is needed to determine the treatment objectives in a patient with lumbosacral agenesis. Important treatment measures include muscle strength and sensorial examination of lower extremities, periodic monitoring of spine for scoliosis and analysis of functions of internal organs, particularly urinary system. At least one intravenous pyelogram must be performed to examine the urinary system abnormalities. [5, 6, 8]

The most important indicator for walking potential is knee flexion contracture. Severity of popliteal web and accompanied contractures are directly associated with quadriceps muscle strength. Knee flexion contractures associated with popliteal web are the most difficult orthopaedic deformities to treat in these cases. Subtrochanteric amputation or knee disarticulation is recommended particularly for cases lack of quadriceps muscle strength [8–10]. Knee disarticulation may be preferred to subtrochanteric amputation to protect the body image, facilitate the application of prosthesis and ensure the functionality of residual lower extremity. Some authors recommend the aggressive orthopaedic approaches but also advise against the difficulty of controlling the knee flexion contractures [9]. Ambulation potentials of cases should also be considered when deciding to treatments to be administered. Cases with thoracal and upper lumbar involvement cannot walk and have to use the wheelchairs. Ambulation in the home may be possible for cases with middle lumbar level but the community ambulation is not possible. Functional ambulation may be possible for cases with lower lumbar level. Cases with lower lumbar and sacral levels may live independently.

## Conclusion

As ambulation is not expected in this case because the robust spinal cord level is T6, the main objective is to ensure sitting stability. Flexion contractures in knees help to sitting stability and thus, increase functionality of patient in daily life. This demonstrates that not every deformity in the body is a pathology that needs to be corrected.
